# The Ecology of Gynecological Care for Women

**DOI:** 10.3390/ijerph110807669

**Published:** 2014-07-31

**Authors:** Chia-Pei Chang, Chia-Lin Chou, Yueh-Ching Chou, Chun-Chih Shao, Irene H. Su, Tzeng-Ji Chen, Li-Fang Chou, Hann-Chin Yu

**Affiliations:** 1 Department of Obstetrics and Gynecology, Taipei Veterans General Hospital, No. 201, Sec. 2, Shi-Pai Road, Taipei 112, Taiwan; E-Mail: jpchang2@vghtpe.gov.tw; 2 Department of Pharmacy, Taipei Veterans General Hospital, No. 201, Sec. 2, Shi-Pai Road, Taipei 112, Taiwan; E-Mails: clchou6@vghtpe.gov.tw (C.-L.C.); ycchou@vghtpe.gov.tw (Y.-C.C.); 3 Department and Institute of Pharmacology, National Yang-Ming University, No.155, Sec.2, Linong Street, Taipei 112, Taiwan; 4 College of Pharmacy, Taipei Medical University, 250 Wu-Hsing Street, Taipei 110, Taiwan; 5 Department of Family Medicine, Taipei Veterans General Hospital, No. 201, Sec. 2, Shi-Pai Road, Taipei 112, Taiwan; E-Mails: jnshao@gmail.com (C.-C.S.); tjchen@vghtpe.gov.tw (T.-J.C.); 6 Department of Reproductive Medicine, University of California, San Diego, 9500 Gilman Dr., La Jolla, CA 92093, USA; E-Mail: hisu@ucsd.edu; 7 Institute of Hospital and Health Care Administration, School of Medicine, National Yang-Ming University, No.155, Sec. 2, Linong Street, Taipei 112, Taiwan; 8 Department of Public Finance, National Chengchi University, No. 64, Sec. 2, ZhiNan Rd., Wenshan District, Taipei 116, Taiwan; E-Mail: lifang@nccu.edu.tw; 9 Taipei Veterans General Hospital Hsinchu Branch, No. 81, Sec. 1, Zhongfeng Rd., Zhudong Township, Hsinchu County 310, Taiwan; 10 Department of Healthcare Management, Yuanpei University, No.306, Yuanpei street, Hsinchu 300, Taiwan

**Keywords:** gynecological, ecology, medical care, national health insurance, utilization

## Abstract

Gynecological care is vital to women’s health but utilization of gynecological care has been seldom addressed. We applied the population-based “ecology model” to demonstrate the utilization of gynecological care of women, with examples from Taiwan. We analyzed the claims data from the cohort datasets within the National Health Insurance Research Database in Taiwan. Women’s utilization of gynecological care in 2009 was computed. Of 1000 women, 319 utilized gynecological care at least once, 277 visited Western medicine clinics, 193 visited physician clinics, 118 visited hospital-based outpatient clinics, 73 visited traditional Chinese medicine clinics, eight were hospitalized, four were hospitalized in an academic medical center, and four visited emergency departments. More than 90% of young and middle-aged women who sought gynecological care visited gynecologist clinics. Elderly women were less likely to utilize gynecological care in all settings of medical care, but were more likely to be attended by non-gynecologists. Young women tended to visit emergency departments. The ecology model highlighted age disparities in women’s utilization of gynecological care in various settings of medical care. Since gynecological conditions were common among women, more attention should be paid on the availability of gynecologists and continuing medical education in gynecological care for non-gynecologists to guarantee women’s health.

## 1. Introduction

Good gynecological care is vital to women’s health. While most gynecological research focused on diagnosis and treatment of diseases, data on women’s utilization of gynecological care are limited. 

Although some nationwide studies has been published to show the utilizations of ambulatory visits [1], emergency department visits [2], and inpatient hospitalizations for gynecological conditions [3], the results are limited in their ability to describe women’s help-seeking behaviors for gynecologic care. In 1961, White *et al*. proposed the ecology model, a patient-centered and population-based approach to illustrate the number of people in the total population who utilized medical services including ambulatory visits and hospitalizations in a given period of time [4]. This approach has been applied to medical services of general population [5,6,7,8] and specific patient groups [9,10,11]. Also, policy makers, medical educators and researchers have repeatedly referred to this model [12,13]. However, the ecology model has not been applied to gynecological care. 

In the current study, we estimate the proportion of adult women who accessed gynecologic care within the framework of nationalized health insurance (NHI) in Taiwan. Specifically, we sought to determine utilization of ambulatory visits, emergency department visits, and inpatient hospitalizations for gynecologic conditions. 

## 2. Materials and Methods

### 2.1. Data Sources 

Since 1995, NHI has provided comprehensive health insurance coverage for all inhabitants in Taiwan. Since then, claims data of health care utilization have been accumulated, and deidentified datasets have been released for research (http://w3.nhri.org.tw/nhird/). In the current study, we obtained a cohort dataset of 1,000,000 people randomly sampled to be representative of NHI beneficiaries (Longitudinal Health Insurance Database 2005: LHID2005). We then analyzed the ambulatory and hospitalization data of women (aged 18 years or older) in 2009. The registry for contracted medical facilities in 2009 (HOSB2009) was also used to know the type of hospitals.

### 2.2. Study Design 

Using White’s model [4], we estimate the proportion of women in Taiwan who had received gynecological care within NHI in 2009. The following algorithm was used. Within the dataset, all diagnoses were coded according to the International Classification of Diseases, Ninth Revision, Clinical Modification (ICD-9-CM). In the current study, we defined gynecological care as a visit/hospitalization with any diagnosis of inflammatory disease of female pelvic organs (ICD-9-CM: 614–616), other disorders of female genital tract (617–629), malignant neoplasm of uterus, part unspecified (179), malignant neoplasm of cervix uteri (180), malignant neoplasm of body of uterus (182), malignant neoplasm of ovary and other uterine adnexa (183), malignant neoplasm of other and unspecified female genital organs (184), uterine leiomyoma (218), other benign neoplasm of uterus (219), benign neoplasm of ovary (220), benign neoplasm of other female genital organs (221), carcinoma *in situ* of cervix uteri (233.1), carcinoma *in situ* of other and unspecified parts of uterus (233.2), carcinoma *in situ* of other and unspecified female genital organs (233.3), or ovarian dysfunction (256). The following estimates for gynecological conditions in different types of setting were computed:

1. Gynecological care utilization: How many women utilized gynecological care within NHI at least once?
2. Visits to Western medicine (WM) clinics: How many women visited either physician clinics or hospital outpatient clinics?
3. Visits to physician clinics (WM): How many women visited physician clinics?
4. Visits to hospital outpatient clinics (WM): How many women visited outpatient clinics in any hospital?
5. Visits to traditional Chinese medicine (TCM) clinics: How many women visited physicians of traditional Chinese medicine, either in TCM physician clinics or at outpatient clinics?
6. Visits to emergency departments (ED): How many women visited emergency departments?
7. Admissions to wards in the hospitals: How many women were admitted to wards in any hospital?

Hospitals were further classified into local community hospitals, metropolitan hospitals, and academic medical centers (AMCs). Estimates were generated for each type of hospitals respectively. TCM services were included in the current study in contrast to previous ecology studies [5,6,7] in which complementary and alternative medicine (CAM) services were analyzed. While TCM is only one of the CAM modalities available in Western countries [14], it is an important form of medical care in Taiwan [15] and could be used as a proxy of CAM. In addition, while CAM services in other countries are seldom reimbursed by third-party payers [16], TCM services in Taiwan are part of NHI benefits.

The variable of age was sub-grouped into 18–39 years (young), 40–64 (middle-aged), ≥65 (elderly) respectively. The specialty of providers was divided into gynecologists and non-gynecologists.

### 2.3. Statistical Analysis 

Data computation and descriptive statistical analysis were performed with the Perl programming language, version 5.12.1 (Perl Foundation, Walnut, CA, USA) [17]. Utilization data were displayed after converting the denominator into 1000 women. 

## 3. Results and Discussion

### 3.1. Results 

**Figure 1:**
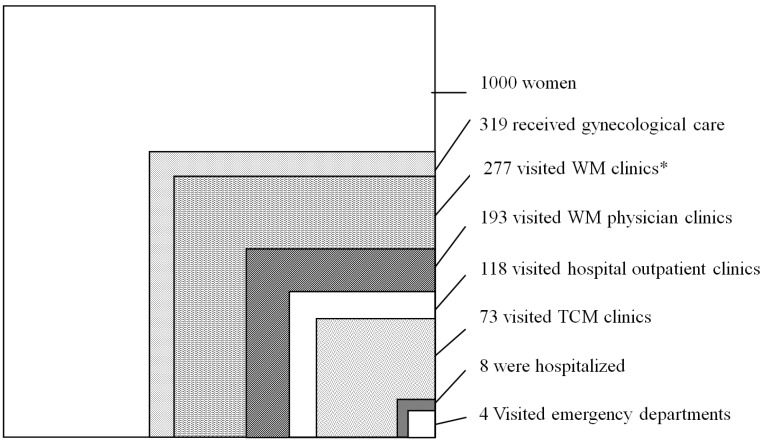
Estimates of women’s utilization of gynecological care in Taiwan in year 2009. Each box does not necessarily represent a subgroup of the larger box (*i.e.*, some values are overlapping). The values are based on 1000 women. Abbreviations: WM *, western medicine; TCM, traditional Chinese medicine.

Note: * WM clinics included physician clinics and hospital outpatient clinics.

In the cohort in 2009, there were 418,066 women, including 178,517 (42.7%) aged 18–39 years, 178,539 (42.7%) aged 40–64 years, and 61,010 (14.6%) aged 65 years and more (Table 1). Over one year, 319 of 1000 women sought medical help for gynecological conditions at least once: 277 visited WM clinics, 193 visited physician clinics, 118 visited hospital-based outpatient clinics, 73 visited TCM physician clinics, eight were hospitalized, four were hospitalized in an academic medical center, and four visited an ED (Figure 1).

Of 277 women seeking gynecological care in WM visits during the year, more than 95% (n = 265) consulted gynecologists rather than non-gynecologists. Of eight women hospitalized for gynecological conditions, seven were treated at gynecological wards and one at non-gynecological wards (Table 2).

Age disparities were observed in health seeking behavior. Elderly women had the lowest utilization of gynecological care. Of 146 elderly women among the 1000 women, only 9% (n = 13) had WM visits for gynecological conditions, 0.2% had ED visits and 0.5% had hospitalizations (Table 1). 

**Table 1:** Estimates of ecology of gynecological care in Taiwan in terms of type of care, stratified by age group.

Category	Number of Women	Received GYN Care	Visits to WM Clinics	Visits to Physician Clinics (WM)	Visits to Hospital Outpatient Clinics (WM)				Visits to TCM Clinics	ED Visits	Hospitalizations			
					Any	LCH	MH	AMC			Any	LCH	MH	AMC
**Number of Women/1000 Women**														
Overall Age	1000	319.3	277.2	192.9	117.8	49.3	45.6	31.8	72.9	4.2	7.9	1.0	3.2	3.6
18–39	427	169.7	144.7	109.8	54.5	27.7	19.4	11.7	47.9	2.6	2.9	0.5	1.2	1.2
40–64	427	135.2	119.8	77.0	55.9	19.5	23.0	17.6	24.6	1.4	4.2	0.5	1.7	2.1
≥65	146	14.4	12.6	6.1	7.4	2.1	3.2	2.5	0.3	0.3	0.7	0.1	0.3	0.4

Notes: GYN, gynecological; WM, western medicine; LCH, local community hospital; MH, metropolitan hospital; AMC, academic medical center; TCM, traditional Chinese medicine; ED, emergency department.

**Table 2:** Estimates of ecology of gynecological care in Taiwan in terms of type of care, stratified by patient age group and provider specialty.

Category	Head Count	Visits to WM Clinics		Physician Clinics (WM)		Hospital Outpatient Clinics (WM)						Hospitalizations							
						LCH		MH		AMC		All		LCH		MH		AMC	
**Number of Women/1000 Women**																			
Provider specialty		GYN	Non-GYN	GYN	Non-GYN	GYN	Non-GYN	GYN	Non-GYN	GYN	Non-GYN	GYN	Non-GYN	GYN	Non-GYN	GYN	Non-GYN	GYN	Non-GYN
Overall Age	1000	264.8	23.7	182.3	15.8	47.7	2.3	43.7	3.0	30.1	3.1	6.6	1.4	0.8	0.2	2.6	0.7	3.2	0.5
18–39	427	142.1	6.4	107.3	4.8	27.3	0.5	19.0	0.6	11.4	0.5	2.5	0.4	0.4	0.1	1.0	0.2	1.1	0.1
40–64	427	112.5	13.8	70.5	9.2	18.6	1.3	22.1	1.7	16.5	2.0	3.6	0.7	0.4	0.1	1.4	0.3	1.8	0.3
≥65	146	10.1	3.5	4.5	1.8	1.8	0.5	2.7	0.7	2.1	0.7	0.4	0.3	0.02	0.04	0.2	0.2	0.3	0.5

Notes: WM, western medicine; LCH, local community hospital; MH, metropolitan hospital; AMC, academic medical center; GYN, gynecologist; non-GYN: non-gynecologist.

In addition, elderly women were more likely to receive gynecological care by non-gynecologists: 28% (3.5/12.6) at all settings of WM visits and 44% (0.3/0.7) in hospitalizations (Table 2).

TCM and ED visits also exhibited age disparities. While 11% (48/427) of young women and 6% (25/427) of middle-aged women had TCM visits, only 0.2% (0.3/146) of elderly women had visited TCM clinics for gynecological conditions. The likelihood of visiting ED by young women (2.6/427) was three times as much as that of elderly women (0.3/146) and almost twice as much as that of middle-aged women (1.4/427) (Table 1).

### 3.2. Discussion

The current study demonstrated that the long established “ecology model” could be well applied to illustrate the women’s utilization of gynecological care. In this model, the hierarchical structure of gynecological care at different settings could be clearly visualized. Monthly estimates, however, were not displayed because gynecological conditions of women were relatively infrequent comparing to general medical conditions of total population.

There were some interesting findings in our current study. First, nearly one-third of women in Taiwan had gynecological conditions leading to at least one ambulatory visit in 2009. Second, while 40% of young women and 30% of middle-aged women utilized gynecological care at least once in a year, only 10% of elderly women received gynecological care. Third, most young and middle-aged women received gynecological care from gynecologists, but non-gynecologists also played a role in providing gynecological care to elderly women.

Overall, most women receiving gynecological care in Taiwan visited gynecologists at all settings of WM clinics. Similar patterns existed in hospitalizations for gynecological conditions. One of the possible reasons is that gynecological care is so distinct from general medical care that non-gynecologists are less well-trained to provide sufficient gynecological care [18]. Another reason is that women might have a preference for gynecologists to provide gynecological care [19].

While elderly people tend to utilize more medical services than other age groups in previous studies [20], elderly women utilized gynecological care less frequently than other age groups in our current study. An American study [1] revealed similar results. One possible explanation is that overall gynecological conditions may become less frequent to elderly women once they are menopausal naturally or after major gynecological surgery, such as hysterectomy [1]. Furthermore, most elderly women are out of the targeted range of age for promotional campaign of cancer screening sponsored by government in Taiwan [21]. For example, breast cancer screening is provided to women aged 45–69 years.

Another feature of elderly women was that they were more likely to receive gynecological care from non-gynecologists. The disparity in provider specialty might reflect that the spectrum of gynecological conditions was different for different age groups of women [22]. Gynecological conditions that elderly women encountered might be either less specific (e.g., gynecological cancers initially presenting with intermittent vaginal spotting) or less severe (e.g., uterine prolapse with urinary incontinence) that elderly women did not feel an urgent need to seek help from specialists. Since elderly women are less mobile, they might turn to non-gynecologists because of better accessibility.

The disparity in TCM utilization for gynecological care among different age groups was compatible with elderly women’s low utilization of gynecological care in every settings of medical care, according to the results of our current study. Besides, young and middle-aged women were more likely to have some common gynecological conditions, such as menstrual disorder [23], for that TCM physicians are often consulted [15]. Finally, female cancers were more prevalent in elderly women [24], and management of female cancers usually required serial examinations or surgical procedures by WM gynecologists.

Although women infrequently visit emergency department for gynecological conditions, younger woman had relatively higher frequencies. Studies in the United States had similar findings [1,2]. It has been reported that there might be differences in incidence of gynecological disorders, with higher rates of pelvic inflammatory diseases and sexual transmitted diseases among the youngest women [2]. 

There were some limitations to the current study. First, we did not examine the spectrum of diagnoses encountered at gynecologic visits. Second, our analysis was based on NHI claims data. The results could not show the utilization of gynecological care that was not reimbursed by NHI. Third, we did not stratify the cohort’s profiles by geographic region because the NHIRD did not contain residence information of beneficiaries. In addition, we used NHIRD 2005 in which 1,000,000 beneficiaries were randomly selected in 2005 and we analyzed the utilization of gynecological care in 2009. Thus patients who died before 2009 may not be included in our study. However, we only analyzed the utilization of gynecological care in the year of 2009, and we did not analyze the survival. Thus the impact should be limited. Finally, the ecology model is only one of methods to illustrate the utilization of medical services. It could not show the frequency of utilization, spectrum of diagnosis, severity of disease and unquantifiable factors that influenced the health seeking behavior.

## 4. Conclusions 

In conclusion, the ecology model highlighted age disparities in women’s utilization of gynecological care in various settings of medical care. Since gynecological conditions were common among women, more attention should be paid on the availability of gynecologists and continuing medical education in gynecological care for non-gynecologists to guarantee women’s health.
